# Predictors of appropriate ICD therapy in patients with implantable cardioverter-defibrillator

**Published:** 2006-01-01

**Authors:** Mohammad Reza Dehghani, Arash Arya, Majid Haghjoo, Zahra Emkanjoo, Mohammad Alasti, Babak Kazemi, Mohammad Hosein Nikoo, Mohammad Ali Sadr-Ameli

**Affiliations:** Department of Pacemaker and Electrophysiology, Rajaie Cardiovascular Medical and Research Center, Iran University of Medical Sciences, Mellat Park, Vali-e-Asr Avenue, Tehran 1996911151, Iran.

**Keywords:** Implantable cardioverter-defibrillator, Appropriate ICD therapy, Coronary artery disease, Dilated cardiomyopathy

## Abstract

**Background:**

Understanding the predictors of appropriate implantable cardioverter defibrillator (ICD) therapy could help to better identify candidates for ICD implantation.

**Methods:**

One hundred and sixty two patients with ICD (111 with coronary artery disease [CAD] and 51 with dilated cardiomyopathy [DCM]) were included in the study. Clinical, electrocardiographic, and ICD stored data and electrograms were collected.

**Results:**

During mean follow up of 15±11 months 54 patients (33%) received ≥ 1 appropriate ICD therapy (AICDT). We used binary logistic regression analysis with forward selection method to find the potential predictors of appropriate ICD therapy after device implantation. Male gender (odds ratio [OR] = 2.76, 95% confidence interval [CI] = 1.1 – 7.1, P=0.021), DCM as underlying heart disease (OR = 4.2, 95% CI = 1.9 – 9.5, P=0.001), and QRS width > 100 ms (OR = 2.58, 95% CI = 1.2 – 5.4, P=0.010) were correlated with increased likelihood of AICDT during the follow up period. In subgroup analysis of the patients with CAD and DCM, QRS duration > 100 ms was correlated with the probability of ≥ 1 AICDT. In our patients indication of ICD implantation (primary versus secondary prevention) did not influence probability of ≥ 1 AICDT (adjusted OR = 1.66, 95% CI = 0.7 – 4.0, Mantel-Haenszel P value P=0.355.)

**Conclusion:**

QRS width could be used as an additional simple risk stratifier beyond EF to identify potential candidates who would benefit more from ICD implantation. This may have practical implications for patient selection especially in developing countries. Indication of ICD implantation (primary versus secondary prevention) did not affect the probability of ≥ 1 AICDT during the follow up period.

## Introduction

Development of implantable cardioverter-defibrillators (ICD) has been a dramatic advancement in the management of patients with ventricular arrhythmias. A major issue in patients with ICD is the high incidence of ICD therapies[1-7], which have a major effect on morbidity and quality of life [4,5]. Knowing the predictors of appropriate ICD therapy could also help to better identify candidates for ICD therapy. This is a retrospective single centre study to identify potential predictors of appropriate ICD therapies.

## Methods

### Patients Population

Between January 2001 and January 2005, 162 patients with coronary artery disease (CAD) or dilated cardiomyopathy (DCM) underwent ICD implantation at our centre. Among these 94 (58%) received single-chamber and 68 (42%) received dual-chamber ICD. The left ventricular ejection fraction (EF) was measured by transthoracic echocardiography. All the patients gave written informed consent for the procedure of ICD implantation. The mean age was 58.2 ± 13.5 years. Table 1 and 2 show the basic characteristics of the patients.

### Implanted ICDs and programming

The implanted devices were manufactured by Medtronic ([GEM-VR, GEM-DR, GEM-II-VR, GEM-II-DR, GEM-III-VR, GEM-III-DR, Marquis-VR, Marquis-DR] Medtronic Inc., Minneapolis, MN, USA) and St. Jude ([Photon-VR, Photon-DR, Photon-μ-VR, Photon-μ-DR, Atlas-VR, Atlas-DR, Epic-VR, and Epic-DR] St. Jude Medical Inc. Sylmar, CA, USA). In implanted devices all the detection and discrimination criteria were activated with the nominal values. In all the devices we defined ventricular fibrillation zone (300 ms) plus one VT zone (400 ms). If the patient had an episode of spontaneous or induced sustained monomorphic VT slower than 370 ms we extended the VT zone to VT cycle length plus 40ms. In the VT detection zone the first therapy was three antitachycardia bursts pacing. We used the nominal values of the ICDs for the duration and tachyarrhythmia detection criteria.

### ICD Data Storage and Retrieval

After ICD implantation the patients were followed on a regular basis (3 months) and upon receiving high voltage therapy in our outpatient ICD clinic. The devices were interrogated at each session and the complete set of data (including intracardiac electrograms) was recorded on floppy diskettes. The summary of the episodes were also recorded in the patient’s file. The floppy diskettes were used in this study to retrieve all spontaneous sustained arrhythmia episodes resulted in ICD therapy. These episodes resulted in ICD therapies, studied by two independent electrophysiologists (AA and MRD) to define the diagnosis. In case of discrepancy in diagnosis the final analysis of the arrhythmia episode was made by a consensus of three electrophysiologists (AA, MRD, and MH). Beside from diagnosis, the time of arrhythmia after implantation and the mode of therapy were recorded.

### Definitions

Appropriate ICD therapy was defined as an antitachycardia pacing or shock therapy for ventricular tachycardia or fibrillation. Indication of ICD implantation was defined as secondary prevention (n=117) in patients who had experienced aborted sudden cardiac death, sustained ventricular arrhythmia, or syncope (whose electrophysiologic study [using three basic drive cycle lengths of 600, 500 and 400 with up to three premature extra-stimuli from right ventricular apex and/or outflow tract] who showed inducible sustained hemodynamically unstable ventricular arrhythmias). The indication of ICD implantation in all the other patients (33 patients with CAD without history of syncope had left ventricular ejection fraction < 40%, nonsustained ventricular tachycardia on Holter monitoring, and inducible sustained hemodynamically unstable ventricular arrhythmia during electrophysiologic study; and 13 asymptomatic patients with DCM with nonsustained ventricular tachycardia during Holter monitoring who had inducible sustained hemodynamically unstable ventricular arrhythmia during electrophysiologic study) was categorized as primary prevention [6].

### Statistics

Variables are expressed as mean ± SD, and percentage. Differences in frequency of characteristics were assessed by independent sample student’s t-test for continuous variables. Chi-square statistics (or fisher’s exact test if applicable) used for discrete variables. We used binary logistic regression analysis with forward selection method to find the potential predictors of appropriate ICD therapy after device implantation. A two-tailed p-value < 0.05 was considered statistically significant. We used SPSS® 13.0 (SPSS Inc. Chicago, IL, USA) for data storage and analysis.

## Results

### Baseline Characteristics

One hundred sixty two patients (123 men) with ICD were followed for a mean of 15±11 months. Table 1 and 2 show the baseline characteristics of these patients. We compared patients’ characteristics between different underlying diseases (Table 1). We took into account these differences in subsequent statistical analysis to find potential predictors of appropriate ICD therapy. Among 49 patients who received ICD as primary prevention, 20 (41%) received appropriate ICD therapy. Among 113 patients who received ICD as a secondary preventive measure 34 (30%) received appropriate ICD therapy (unadjusted odds ratio [OR] = 1.74, 95% confidence interval [CI]: 0.8 - 3.6, Mantel-Haenszel P value P=0.14). We adjusted this analysis for left ventricular EF, QRS width, gender, and underlying heart disease which resulted in adjusted OR = 1.66, 95% CI = 0.7 – 4.0, Mantel-Haenszel P value P=0.355. During the same period 30.2% (n = 49) of patients received inappropriate ICD therapy. Twenty seven out of 54 patients (50%) who received appropriate ICD therapy received also inappropriate ICD therapy. The rate of inappropriate ICD therapy among patients who did not receive appropriate ICD therapy (n = 108) was 20.4%. Atrial fibrillation/tachycardia, sinus tachycardia, and oversensing were the most common causes of inappropriate ICD therapy in our patients.

### Predictors of ≥ 1 Appropriate ICD therapy

During the follow up period, 54 patients (33%) received appropriate ICD therapy (Table 2). There were several differences between patients with and without ≥ 1 appropriate ICD therapy. We used these variables as covariates to find the predictors of appropriate ICD therapy during the follow up period. We also included all the other parameters which showed a P<0.3 during bi-variable correlation with probability of ≥ 1 appropriate therapy a binary logistic regression analysis model.

During binary logistic regression analysis QRS width > 100 ms (P=0.003), male sex (P = 0.021), and DCM as underlying heart disease (P = 0.001) were correlated with ≥ 1 appropriate ICD therapy in all patients (Table 3). We chose the subgroups of left ventricular ejection fraction and QRS duration for this analysis based on the median values in the study population. Other factors including age, left ventricular EF, baseline medical therapy (including amiodarone), and indication of ICD implantation failed to correlate with the probability of ≥ 1 appropriate ICD therapy during the follow up period (all Ps > 0.05).

### Number of Appropriate ICD therapies

During our follow up period the above mentioned patients received mean number of 17±29 (range 1 – 132) appropriate ICD therapy. Among these the number of appropriate ATP was 11.9±28 (range 0 – 131) and the number of appropriate shock therapy was 5.1±9.9 (range 1 – 56). The success rate of ATP therapy among the episodes in the VT detection zone was 88%. However, as our study is retrospective it is possible that after first appropriate ICD therapy, electrical and/or medical treatment was adjusted or optimized to prevent new VT recurrences. Therefore, to minimize this effect we just assessed the predictors of ≥ 1 appropriate ICD therapy rather than total *number* of appropriate ICD therapies.

During subgroup analysis in patients with CAD and DCM, QRS duration > 100 ms was correlated with the probability of ≥ 1 appropriate ICD therapy during binary logistic regression (P = 0.006 and P = 0.003, respectively).

## Discussion

The main findings of this retrospective study are 1) QRS width might be a useful risk stratifier (beyond ejection fraction) in patients with CAD and DCM; 2) adjunctive amiodarone therapy in our patients failed to decrease the incidence of ≥ 1 appropriate ICD therapy; 3) indication of ICD implantation did not influenced the probability of ≥ 1 appropriate ICD therapy during the follow up period. Several points merit consideration. The rate of appropriate ICD therapy was different in our patients compared to the other studies [1,4,7]. This difference can be at least partially explained by the different patients population and the follow up period.

No empiric antiarrhythmic therapy (including amiodarone) is *currently* indicated in patients who received an ICD. These patients frequently receive ICD shocks, which severely impair quality of life. Intravenous amiodarone followed by oral dose, in patients with electrical storm results in successful management of ventricular arrhythmias and possibly a long-term prognosis similar to patients who do not have electrical storm [9,10]. The OPTIC (Optimal Pharmacological Therapy in Implantable Cardioverter) study currently assesses the potential benefit of antiarrhythmic medications in reduction of ICD therapy and electrical storm. In OPTIC the patients are randomized to β-blocker, amiodarone plus β-blocker, or sotalol [9]. Amiodarone may have some other potential benefits in patients with ICDs including the prevention of supraventricular tachycardia, and so may decrease inappropriate ICD discharges; and the prevention of nonsustained but symptomatic ventricular arrhythmias. Further studies are warranted to clarify these issues [11].

Patients with ICD who received it for primary prevention of sudden cardiac death received frequent appropriate ICD therapy. Among our patients the probability of ≥ 1 appropriate ICD therapy among those who received ICD for primary and secondary prevention was comparable. Although this may be partially explained by our restricted criteria of ICD implantation for primary prevention (see definition) it also highlights the importance of implementing guidelines of primary prevention of sudden cardiac death in daily clinical practice. However, it should be mentioned that practically for the majority of the countries (especially the developing countries) and health care providers it is simply economically impossible to follow all the indications derived from the recent primary prevention ICD-trials in which the major risk stratifier is EF [12]. Therefore, further risk stratification beyond EF is highly desirable and necessary [12].

Several epidemiological studies showed that QRS duration (both its absolute value and its dynamic changes) predicts sudden and all-cause mortality [13-16]. These studies have shown that the rate of sudden and non-sudden cardiac mortality increases sharply as QRS duration rises above 120 ms. This effect is observed both in patients with and without bundle branch block. Our finding also showed that QRS width ≥ 100 ms (median value in our study population) is associated with increased incidence of appropriate ICD therapy (Table 3). However, a recent comparison of QRS duration with microvolt T wave alternans showed a false negative rate of narrow QRS complex (10.2%) among MADIT II like patients which could limit its value as a risk predictorc [17].

Finally, only 57% of our patients received beta-blocker therapy. There is substantial evidence that beta blocker therapy has positive effects on morbidity, and mortality, in patients who have been diagnosed with heart failure and/or CAD. Beta blockers should be considered a cornerstone of therapy for these patients [18-20]. Therefore although in our study beta-blocker therapy failed to reduce the incidence of appropriate ICD therapy, beta-blockers should be administered to all patients with CAD and DCM who have ICD unless an absolute contraindication is present. This will decrease the morbidity and mortality in these patients as the beneficial effect of beta-blockers in these patients is additive to the effect of ICD.

### Study limitation

Although we showed that (1) the adjunctive amiodarone therapy do not reduce the incidence of ≥ 1 appropriate ICD therapy and (2) beta-blockers failed also to reduce it, our study was retrospective and non-randomized. Before making any conclusion from our data we have to wait for results of randomized studies such as OPTIC.

## Figures and Tables

**Table 1:**
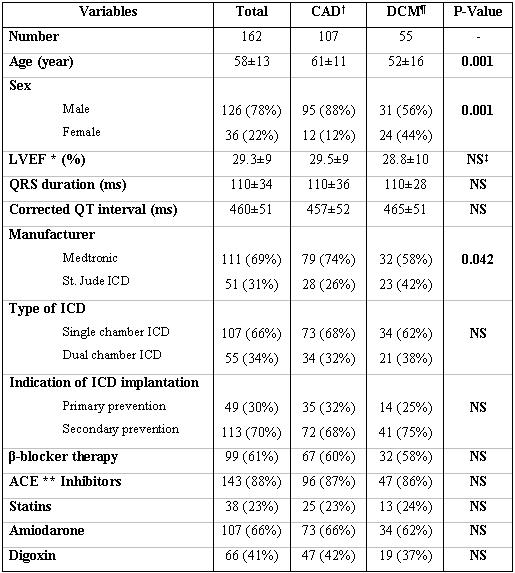
Baseline characteristics of patients based on underlying heart disease

*: **LVEF:** Left ventricular ejection fraction

**: **ACE:** Angiotensin converting enzyme

‡ **CAD:** Coronary heart disease

¶ **DCM:** Dilated cardiomyopathy

† **NS:** non-significant

**Table 2:**
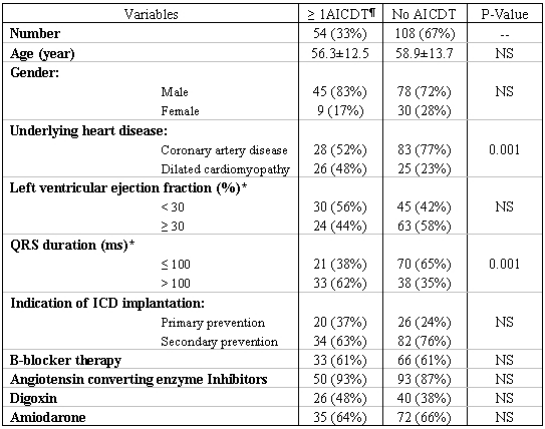
Characteristics of patients based on ≥ 1 appropriate ICD therapy

*: The subgroups of left ventricular ejection fraction and QRS duration are chosen based on the median values in the study population

**: Sustained ventricular arrhythmia requiring ICD therapy

‡ Appropriate ICD therapy

† Patients with sustained monomorphic ventricular tachycardia requiring ICD therapy

**Table 3:**
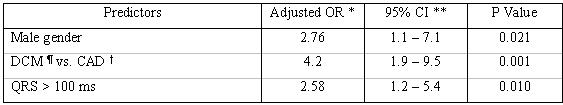
Predictors of appropriate ICD therapy

*: Odds ratio. Each odds ratio is adjusted for the other two predictor variables

**: Sustained ventricular arrhythmia requiring ICD therapy

‡ Confidence interval

† Coronary artery disease

¶ Dilated cardiomyopathy
